# Jin’s three-needle acupuncture technique for chronic fatigue syndrome: a study protocol for a multicentre, randomized, controlled trial

**DOI:** 10.1186/s13063-019-3243-5

**Published:** 2019-03-04

**Authors:** Wenjia Lin, Xin-lin Chen, Qi Chen, Junmao Wen, Xinghua Chen

**Affiliations:** 1 Guangzhou University of Traditional Chinese Medicine, Guangzhou, 510405 China; 2 School of Basic Medical Science, Guangzhou University of Traditional Chinese Medicine, Guangzhou, 510006 China; 3 The First Affiliated Hospital, Guangzhou University of Traditional Chinese Medicine, Guangzhou, 510405 China

**Keywords:** Chronic fatigue syndrome, Jin’s three-needle technique, Acupuncture, Randomised controlled trial

## Abstract

**Background:**

With an unclear pathomechanism, no confirmed treatment regimen has been established for chronic fatigue syndrome (CFS). Acupuncture is applied as an alternative therapy for CFS. As a kind of acupuncture therapy, Jin’s three-needle acupuncture (JTN) has been applied to treat CFS. However, few large-sample randomised controlled trials on JTN treatment for CFS have been reported. We designed this study to evaluate the efficacy and safety of JTN treatment for CFS.

**Method/design:**

This study is a multicentre, single-blind, randomised controlled trial. Patients who meet the inclusion criteria will be recruited and randomly assigned to either the JTN treatment group or the basic acupuncture group. Both interventions will be conducted for five consecutive days per week and last for 2 weeks. The primary outcome is the effective rate based on the 14-item Fatigue Scale (FS-14) score. Other outcome measures include the Fatigue Assessment Scale (FAI), the Depression Status Inventory (DSI), and the Self-rating Anxiety Scale (SAS). Plasma adrenocorticotropic hormone (ACTH), plasma cortisol, and serum levels of IL-2 and IFN-γ will also be measured in this study. Adverse events will be observed and recorded for the safety evaluation.

**Discussion:**

This study may help to identify the efficacy and safety of JTN acupuncture treatment for CFS.

**Trial registration:**

Chinese Clinical Trial Registry, ID: ChiCTR-IOR-17011009. Registered on 29 March 2017.

**Electronic supplementary material:**

The online version of this article (10.1186/s13063-019-3243-5) contains supplementary material, which is available to authorized users.

## Background

Chronic fatigue syndrome (CFS) was first named by the United States Centers for Disease Control and Prevention (CDC) in 1988 [1]. CFS is characterised by chronic fatigue and a series of atypical symptoms, such as muscle weakness, cognitive impairment, emotional stress, digestive disorders, depression, and injured immune function.

Based on recent reports, between 836,000 and 2.5 million individuals are affected by CFS in the US [2, 3]. The overall estimated minimal yearly incidence provided by another investigation conducted in England was 0.015% [4]. Official epidemiological data for CFS were not available in mainland China, but an investigation in Hong Kong reported that approximately 0.6 million adults were influenced by CFS, particularly among ageing, female, and low-socioeconomic status groups [5]. Furthermore, with medical costs estimated between US$2342 to US$8675 for each patient, CFS is a stressful economic burden on the patients, their families and society [6].

However, with obscure aetiology [7], neither established diagnostic tests nor any FDA-approved drugs are available for CFS. The use of rintatolimod and rituximab as well as cognitive behavioural therapy (CBT) and graded exercise therapy (GET) may be beneficial for CFS patients [8–10], but the evidence of their effectiveness is still limited [11–15]. Findings from studies add to the evidence that straightforward, non-pharmacological therapies may be helpful in treating CFS [11]. Thus, complementary and alternative medicine for CFS may have considerable therapeutic potential for CFS patients.

As a non-pharmacological therapy, acupuncture shows benefits in improving the symptoms of CFS patients [16–19]. Additionally, for the limited quality of methodology, high-quality randomised controlled trials (RCTs) are still needed to assess the efficacy of acupuncture for treating CFS [18, 20].

We designed this study to use two forms of acupuncture. One form is basic acupuncture. This treatment for CFS is initiated from ‘*Science of Acupuncture and Moxibustion (9th edition, 2012)*’, a widely used textbook compiled by experts from 20 universities organised by the State Administration of Traditional Chinese Medicine (NATCM) in China [21]. Previous RCTs have proven the efficacy of basic acupuncture as a kind of baseline therapy [22–24]. In one study, the researcher reported an effective rate of 63.3%. Based on symptom evaluations, nine patients in the basic acupuncture group reported that their symptoms improved by more than two thirds compared with baseline symptoms. Ten patients reported that their symptoms were improved by a third. The 14-item Fatigue Scale (FS-14) also showed statistical significance via the paired- sample *t* test. The author considered this therapy mechanism for the regulation of liver and renal visceral functions, and as a benefit to the spleen *qi* [22]. Further, the chosen points, such as ST36, SP6, GV20, BL23, and CV4, were the top five acupoints selected for CFS based on a systematic review [20].

The other treatment is Jin’s three-needle acupuncture (JTN) treatment, a school of acupuncture treatment originating in South China. JTN has been widely used to improve sub-healthy conditions or treat diseases such as depression [25]. A previous RCT has noted that JTN treatment may improve somatic and mental fatigue in CFS patients. However, the sample size was small, and this study mainly recruited patients aged 18–30 years [22]. There is still a lack of RCTs for the study of JTN treatment on CFS.

We designed an RCT to compare basic acupuncture treatment and JTN treatment. The trial aims to identify the efficacy and safety of JTN treatment for CFS patients.

## Methods/design

**Fig. 1:**
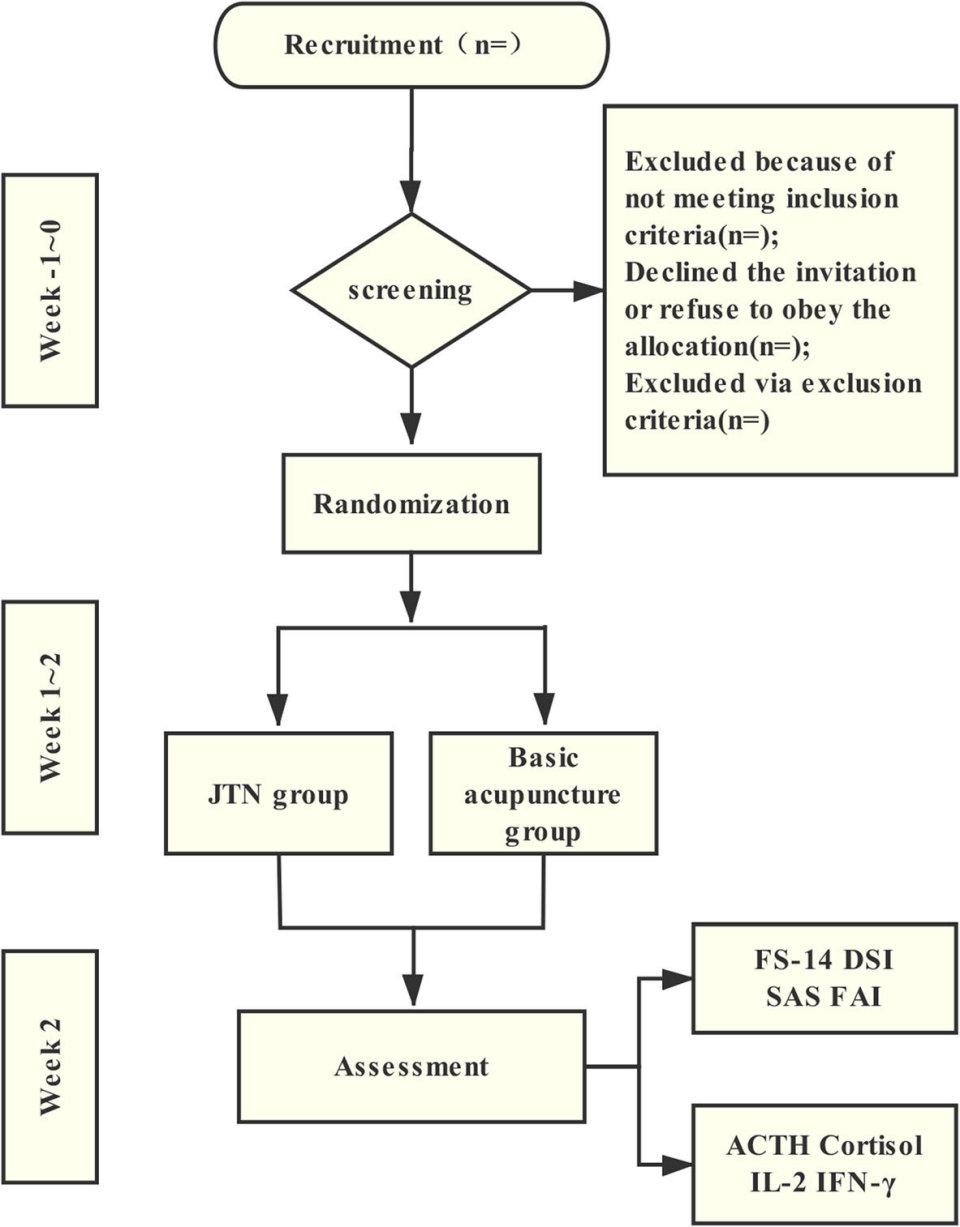
Trial flowchart

**Fig. 2:**
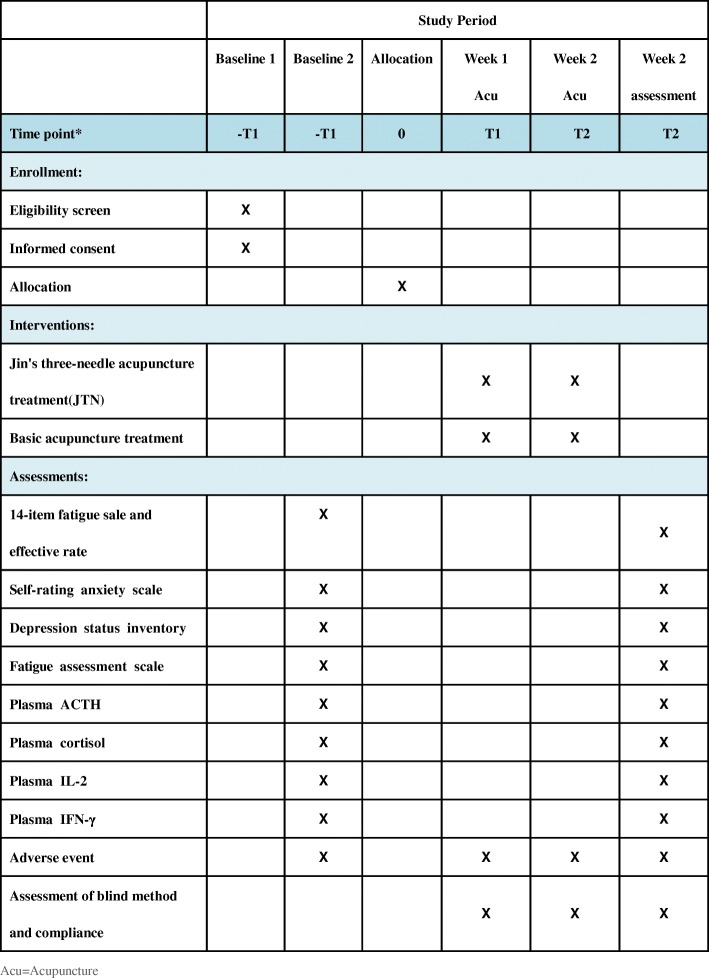
Schedule of enrolment, interventions, and assessments according to the Standard Protocol Items: Recommendations for Interventional Trials (SPIRIT) guidelines

This study is a multicentre, single-blind, RCT conducted in Guangzhou, Guangdong Province, China. The study centres include the First Affiliated Hospital of Guangzhou University of TCM, the Third Affiliated Hospital of Sun Yat-sen University, and Guangzhou Dongsheng Hospital. All study settings are governmental hospitals, and two are academic hospitals. The CFS patients who match the inclusion criteria will be recruited and randomly assigned to the JTN group and the basic acupuncture group. The allocation ratio is 1:1. All patients will be asked to sign the Consent Form before the screening. These two interventions will be given five times per week for 2 weeks. Data will be managed by statisticians and blind evaluators during the study. This trial protocol has been approved by the Ethics Committee of the First Affiliated Hospital of Guangzhou University of TCM (No. ZYYECK2016–023) and registered in the Chinese Clinical Trial Registry (ChiCTR-IOR-17011009).The trial flowchart and study design schedule are presented in Fig. 1 and Fig. 2, respectively. The Standard Protocol Items: Recommendations for Interventional Trials (SPIRIT) Checklist is given in Additional file 1.

### Sample size

The sample size was calculated via the effective rate based on the FS-14. In some previous acupuncture studies for CFS, researchers adopted the FS-14 score as one of the primary endpoints. The FS-14 score was applied with the formula:

$$ \left[\left(\mathrm{Value}\ \mathrm{pretreatment}-\mathrm{Value}\ \mathrm{posttreatment}\right)/\mathrm{Value}\ \mathrm{pretreatment}\times 100\%\right)\Big] $$

to calculate the effective rate [26]. Thus, based on this method and data from our preliminary study, we assumed the effective rate for the control group to be approximately 64% and the effective rate of JTN treatment to be approximately 86%. Therefore, based upon a two independent proportions power analysis conducted by an independent statistician via PASS 11.0, approximately 80 participants in each group will be needed to detect a statistically significant difference, with a significance level of 5% and a power of 90%. To allow for a predicted dropout rate of 15%, a sample size of 188 participants in total will be sufficient to detect a clinically important difference in this trial.

### Participants

Inclusion criteria:(1) patients who meet the diagnostic criteria of CFS (published in 1994, by the Centers for Disease Control) [1]; (2) patients aged between 20 and 55 years; (3) patients who volunteered to participate in the study and signed Informed Consent Forms; (4) patients who did not receive any related therapy, such as pharmacotherapy and psychotherapy within 1 month; and (5) patients who had at least a high-school diploma and had the ability to understand the scale.

Exclusion criteria:(1) pregnant and lactating women, and women who have fertility intention within 3 months; (2) patients who have contagious disease, mental illness, cardiovascular diseases, or primary diseases of the liver, kidney, and haematopoietic and endocrine systems; (3) patients with haemorrhagic disease or a bleeding tendency; (4) patients who experienced trauma, fever, or allergic history in the recent 2 weeks; (5) patients who faint during acupuncture or from the sight of blood; and (6) patients who are participating in other clinical trials concurrently.

### Recruitment procedures

The study centres will recruit participants via advertisements on the Internet and posters on bulletin boards at the hospitals. The contents of the recruitment advertisement include a brief introduction to CFS, the inclusion criteria, the intervention, and the contact information of the researchers. If a patient is interested in participating in the trial, then they will be invited to consult the researchers. Once the patient signs the Informed Consent Form, they will be screened. Patients who match the diagnostic and inclusive standards will formally enter the study. The following basic personal information will be collected: sex, age, Body Mass Index (BMI), educational background, occupation, marital status, related past health history, etc. These details about the participants will be maintained by the Data Monitoring Committee (DMC) and will never be revealed to any individual or organisation relevant to the study.

### Ethical considerations

The study protocol was approved by all relevant Institutional Review Boards: the First Affiliated Hospital of Guangzhou University of TCM, the Third Affiliated Hospital of Sun Yat-sen University, and Guangzhou Dongsheng Hospital. The researchers will obtain written informed consent from each participant before screening. The informed consent form (in Chinese) is given in Additional file 2.

### Intervention

Both treatments will be conducted for five consecutive days per week and last for 2 weeks. We will use No. 30 disposable acupuncture needles (size: 0.32 × 40 mm, 0.30 × 25 mm; Suzhou Tianxie Acupuncture Instruments Co., Ltd., HuangqiaoQingtai, Suzhou, China) for the intervention. The quality of the needles will be checked before conducting the treatment to ensure safety.

The practitioners of this trial have more than 3 years of clinical experience and have participated in a training session to ensure that the treatments will be administered consistently. The patient may make an appointment with the physician before starting the treatment, and confirmation will be made by a phone call to enhance compliance.

### The JTN group

According to the JTN theory, every point group is compatible with some kind of disease or a series of symptoms. Thus, in the treatment group, two groups of points are chosen: ‘the group for fatigue’ and the ‘three-foot-point group’. The former consists of the ‘*Sishen* points’ (also named ‘*Sishen zhen*’, a special point), PC6, and ST36, and the latter consists of ST36, SP6, and LR3 [27, 28]. Based on the evaluation of acupuncture literature, the final point prescription is the combination of the two groups plus another three points: GV20, RN4, and BL23.

During the course, patients randomly assigned to the treatment group will be treated while in the lateral position. The patients may lie on their left side in the first week; then, they will turn to the right lateral position in the second week, or vice versa. The position of the patients will be recorded during the course.

Among the selected points, PC6, ST36, SP6, and LR3 will be needled unilaterally based on the position of the patient. PC6 and LR3 will be needled at 45–90° to the body surface and inserted at a depth of 1.0 *cun*. ST36 and SP6 will be needled at 90° to the body surface and inserted at a depth of 1.5 *cun*. RN4 will be needled at 45–90° towards the bottom and inserted at a depth of 1 *cun*. BL23 will be needled 45–90° downward at a depth of 1–1.5 *cun*. On the head, GV20 will be needled at 15–30° to the body surface and inserted at a depth of 0.5 to 1.5 *cun*.

Finally, at the *Sishen* points, the needles will be inserted 1 *cun* from the four sites obliquely sideways from GV20 at an angle of 15–30° to the body surface. The needling sensations felt by the patient, ‘*Deqi*’, will be achieved by lifting, thrusting, and twirling the needles during the needling the points. The needles will be retained for 30 min for each treatment session. The needles at the *Sishen* points, PC6 and ST36, will be gently twirled every 10 min to achieve and enhance the sensation according to the JTN theory.

### The basic acupuncture group

In the first 20 min, the participants appointed to the control group will be treated in the supine position. At RN4 in the abdominal region, the needle will be tilted at 90° to body surface and inserted 1.5 *cun*. At bilateral LR3 in the foot region, the needle will be tilted 45 to 90° and inserted 0.5–1 *cun*. At bilateral ST36 and SP6, the needle will be tilted 90° to the body surface and inserted 1–1.5 *cun*. Then, in the next 10 min, the participants will be treated in a prone position. For GV20 on the head, the needle will be tilted anterior to the body and inserted 0.5 to 1 *cun*. At bilateral BL23 in the back region, the needle will be tilted 45 to 90° towards the bottom and inserted 1 to 1.5 *cun*. The needles will be twirled to enhance the sensation during treatment. If a patient feels pain, then we will slightly change the depth or angle of the needle.

### Concomitant treatments

The patients in the two groups will be discouraged from any additional specific complementary treatments related to CFS throughout the trial, including moxibustion, herbal medicine, massage, etc. If a patient needs medications, such as antidepressant drugs and anxiolytics, or to undertake mind-cure, then the relevant information will be recorded in the Case Report Form for that patient. Two researchers, blinded to the treatment, will assess whether to adopt the data.

### Outcome measures

As the CFS diagnostic criteria are mainly based on the patient’s symptoms, we considered symptom improvement as the primary outcome. Thus, the patient-reported outcome (PRO) method was adopted in this trial. The primary outcome was the effective rate based on the FS-14 score. The FS-14, which is used to evaluate the fatigue level of the patient, reflects the severity of fatigue from two different angles: physical fatigue and mental fatigue [29, 30]. A higher score suggests more serious fatigue. The FS-14 score will be applied with a formula, namely:

$$ \left[\left(\mathrm{Value}\ \mathrm{pretreatment}-\mathrm{Value}\ \mathrm{posttreatment}\right)/\mathrm{Value}\ \mathrm{pretreatment}\times 100\%\right] $$

to calculate the effective rate. If the integral is reduced by ≥ 60% after treatment, then the treatment will be marked as a notable effective. If the integral is reduced by < 60% but ≥ 30%, then the treatment will be considered as effective. If the integral is reduced by < 30%, then the treatment will be regarded as ineffective [26].

The secondary outcomes include the Fatigue Assessment Scale (FAI), Depression Status Inventory (DSI), and Self-rating Anxiety Scale (SAS): (1) The FAI, a 29-item scale, with scores ranging from 1 to 7 for each choice, evaluates four dimensions of fatigue: fatigue severity (11 items), the sensitivity of fatigue under particular circumstances (six items), associated consequences of fatigue (three items), and the response to sleep (two items). A higher score indicates greater problems with fatigue [31, 32]; (2) The DSI, consisting of 20 questions, is used to evaluate the depressive state of the patient in the past week. This assessment reflects the emotional, somatic, psychomotor retardation, and mental disorders of depression. The DSI is especially suitable for general hospitals to find patients with depression [33]; and (3) The SAS, containing 20 questions, is used to evaluate the level of anxiety of a patient at 1 or 2 weeks before taking the test. The assessment is based on scoring in four groups of manifestations: cognitive, autonomic, motor, and central nervous system symptoms [34].With scores ranging from 1 to 4, a higher SAS or DSI score indicates a higher risk of developing anxiety and depression.

In addition, plasma adrenocorticotropic hormone (ACTH), plasma cortisol, and serum levels of interleukin-2 (IL-2) and interferon-gamma (IFN-γ) will be examined to determine whether the immune and hypothalamic-pituitary-adrenal axis (HPA) functions of CFS patients will be changed by JTN treatment.

Previous studies have indicated the hypoactivity of the HPA axis in CFS [35]. In a study focusing on basal circadian ACTH and cortisol secretion in patients with CFS, the mean basal level of plasma ACTH in the patients was 1.8 ± 0.2 pM, while that in the controls was 2.4 ± 0.5 pM. The average baseline cortisol secretion was 3.1 ± 0.5 μg/dl in patients with CFS and 2.7 ± 0.4 μg/dl in the controls. Researchers have considered that basal hypercortisolaemia results from the resiliency of the HPA axis [36]. In this study, we will measure plasma ACTH and cortisol levels to study changes in HPA function after acupuncture treatment. We assume that the basal level of the two indicators will be increased after treatment.

Previous studies have reported Th1 cytokine abnormalities in CFS, and IFN-γ was found to be increased in CFS patients, while IL-2 was reported with mixed findings [37–39]. According to Fletcher [39], plasma concentration of IFN-γ in CFS cases was 3.1 (0.1–11.8) pg/ml, while that in controls was 2.6 (1.2–10.6) pg/ml. Plasma IL-2 was 2.3 (1.4–5.4) pg/ml in CFS cases and 2.5 (2.1–3.5) pg/ml in the controls. In this study, IL-2 and IFN-γ will be measured to detect whether the level of the two indicators will be reduced after treatment.

All scale and plasma indicators will be assessed before treatment and immediately after treatment.

### Adverse events and safety monitoring

Adverse events will be recorded and managed by researchers within 24 h. There will be at least one physician to evaluate and manage the adverse events at each centre. Serious adverse and unexpected events will be reported to the Ethics Committee and other supervising departments. The subjects may leave the trial at their own discretion, or the physicians will determine whether the patient will continue or terminate the study. However, the patient will be followed up until they are in a stable condition following the adverse event.

The primary investigator will review all adverse events periodically, and the Ethics Committee and DMC will have access to the interim results. If necessary, a meeting will be held to reappraise the benefits and risk of this trial.

The possible adverse events related to acupuncture treatment include fainting during acupuncture treatment, nausea, haematoma formation, local infection, injury to the viscera or the peripheral nerves, stuck needle, and needle breakage. Adverse events will be evaluated based on the frequency, severity and symptoms of the patients throughout the trials. Patients who are intolerant to the acupuncture treatment should be removed from the trial.

### Randomisation

A total of 188 participants who meet the selection criteria will be randomly allocated into the JTN group and the basic acupuncture group. A statistician blinded to the treatment and data collection will use Strategic Applications Software (SAS, version 9.1.3, SAS Institute Inc., Cary, NC, USA) to generate the random allocation sequence. The group name will be written on a card and sealed in an opaque envelope. The sequence numbers will be written on the envelopes, and the envelopes will be numbered sequentially. Random allocation will be performed for participants who meet all selection standards and signed the Consent Form. The research coordinator will allot participant identification codes and record the codes in the Case Report Forms. The acupuncturists will sequentially open random-allocation envelopes and allocate the participants accordingly. The details (such as name, date of birth, date of inclusion, etc.) of newly included participants will be recorded before randomisation. The opened envelopes will be stored separately in locked cabinets.

### Blinding

The participants will be blinded in this trial. The coordinator will send the study number and group allocation to the acupuncturist via Email. The records of the acupuncturist will be stored in a locked briefcase that will only be available for the acupuncturists and cooperative researchers. Only the acupuncturists and the coordinators will know the group allocation of each participant. The researchers and acupuncturists must not discuss related information with the participants. As patients in the two groups will adopt different positions, these individuals will not be treated in the same place after randomisation. Acupuncturists will inform the patients about the location of the treatment rooms prior to each treatment session. Therefore, the chance of acquaintance between patients in the two groups will be reduced as much as possible.

Unblinding is permissible when a participant leaves the trial at their own discretion or the physicians determine that they should terminate the trial. The blinding will be monitored and assessed by independent statisticians responsible for data monitoring and by the Ethics Committee of the study centre. After receiving permission from the supervisors, the acupuncturists may reveal the allocated intervention to the patient who dropped out.

### Data collection and statistical analysis

Acupuncturists will collect the data using Case Report Forms. Two independent researchers will then input the data into the database. All analyses will be carried out under the guidance of independent data monitors to ensure the safety and reliability of the data. For participants who drop out, we will record their number and the reason for termination, especially for adverse events.

All enrolled patients will be included in the primary analysis conducted in accordance with the intention-to-treat (ITT) principle. The data will be analyzed by SPSS for Windows version 19.0. The statistical significance is defined as a two-sided *P* value of ≤ 0.05. The baseline characteristics will be reported as the means ± standard deviation (SD). For comparison of baseline materials, a chi-squared or Fisher’s exact test will be used for categorical variables, Student’s *t* test will be used for normally distributed data and the Mann-Whitney *U* test will be used for non-normally distributed data. The scores of each rating scale and laboratory index before the intervention will be compared between the groups via Student’s *t* test or the Mann-Whitney *U* test. For the comparison within groups, the changes in the scores and indexes from baseline to endpoint will be assessed by a paired *t* test or the Wilcoxon rank-sum test. For the difference between groups, we will use Student’s *t* test or the Mann-Whitney *U* test. For the safety analysis, the adverse events will be listed and analyzed using a chi-squared or Fisher’s exact test. For participants who discontinue or deviate from intervention, listwise deletion or multiple imputation will be used.

### Composition of the Data Monitoring Committee (DMC)

The DMC of the study centre comprises experienced experts on experimental statistics in the Guangzhou University of Traditional Chinese Medicine (GZUTCM). The DMC periodically reviews the trial on issues such as the execution of the trial, collection of the data, allocation concealment and personal privacy protection. This committee will also provide advice on the modification or termination of the trial. The DMC is independent from the sponsor and has no competing interests.

### Public access to the protocol and data

In accordance with the data-sharing policy of CHICTR (http://www.chictr.org.cn), the data from this study will be uploaded to the ResMan database within 6 months after the trial is completed. The data will become available to outside researchers at the conclusion of the trial and following the publication of the main study findings as a limited-use dataset with documentation. The study participants were informed about data sharing with external investigators in the Consent Forms. All outside investigators will be asked to sign a data-use agreement to protect study participant confidentiality.

## Discussion

To our knowledge, this study is the first multicentre, single-blinded, and randomised acupuncture study applying JTN to treat CFS patients.

JTN is a kind of acupuncture therapy characterised by its special way of choosing treatment points. An acupuncture prescription is made up of one or more acupoint groups. Each group consists of acupoints that are influential points, empirical points, or local points. The combination of these points may generate an obvious curative effect compared with a normal mixture of ‘cookbook’ points. Thus, JTN offers the advantages of less acupoint selection with a distinct effect.

Based on the theory of traditional Chinese medicine, the points we chose are for energy balancing, which is necessary for improving the symptoms of CFS. Points such as PC6, ST36, SP6, and LR3 are regional distal points that act on an entire area to improve the state of energy imbalance from which the patient suffers. PC6, ST36, and the empirical point named the *Sishen* point also comprises a point group aimed at relieving fatigue. This group may help to tranquilise and free the body from tension. To prove the effect, we expect to gather related evidence via the assessment of the chosen scales and plasma indicators.

Notably, previous researchers have shown that HPA-axis dysfunction plays a major role in CFS development [40], and HPA-axis hypofunction may lead to inflammation/immune activation. It has been reported that the IL-2 and IFN-γ levels in CFS patients were increased compared to those in the non-fatigued controls [41], which may reflect a change in the Th1/pro-inflammatory immune response and NK cell function. Thus, the plasma ACTH, plasma cortisol, and serum levels of IL-2 and IFN-γ will be examined in this study to determine whether JTN treatment affects the immune and HPA functions of CFS patients.

One of the limitations of this study is that the patients were being followed up only at week 2. But we have trailed some of the patients at 4 weeks posttreatment by assessment of scales. The data of the 4-week follow-up will be collected for further analysis of the long-term effect of JTN.

In conclusion, the results of this study are expected to provide evidence on the efficacy and safety of JTN for CFS. Relevant plasma indicators will be measured to identify whether immune and HPA functions will be changed by acupuncture treatment.

## Trial status

This trial is currently recruiting participants.

## Additional files

Additional file 1: Standard Protocol Items: Recommendations for Interventional Trials (SPIRIT) Checklist. (DOCX 52 kb)

Additional file 2: Informed Consent Form (in Chinese). (PDF 190 kb)

## Abbreviations

CFS: Chronic fatigue syndrome
DSI: Depression Status Inventory
FAI: Fatigue Assessment Scale
FS-14: 14-item Fatigue Scale
HPA: Hypothalamic-pituitary-adrenal axis
JTN: Jin’s three-needle acupuncture
SAS: Self-rating Anxiety Scale
